# Revisiting regional variation in the age‐related reduction in sweat rate during passive heat stress

**DOI:** 10.14814/phy2.15250

**Published:** 2022-04-11

**Authors:** Madison D. Schmidt, Sean R. Notley, Robert D. Meade, Ashley P. Akerman, Maura M. Rutherford, Glen P. Kenny

**Affiliations:** ^1^ 6363 Human and Environmental Physiology Research Unit School of Human Kinetics University of Ottawa Ottawa Ontario Canada; ^2^ Harvard T.H. Chan School of Public Health Harvard University Boston Massachusetts USA; ^3^ Clinical Epidemiology Program Ottawa Hospital Research Institute Ottawa Ontario Canada

**Keywords:** aging, heat, sweat, thermoregulation

## Abstract

Aging is associated with attenuated sweat gland function, which has been suggested to occur in a peripheral‐to‐central manner. However, evidence supporting this hypothesis remains equivocal. We revisited this hypothesis by evaluating the sweat rate across the limbs and trunk in young and older men during whole‐body, passive heating. A water‐perfused suit was used to raise and clamp esophageal temperature at 0.6°C (low‐heat strain) and 1.2°C (moderate‐heat strain) above baseline in 14 young (24 (SD 5) years) and 15 older (69 (4) years) men. Sweat rate was measured at multiple sites on the trunk (chest, abdomen) and limbs (biceps, forearm, quadriceps, calf) using ventilated capsules (3.8 cm^2^). Sweat rates, expressed as the average of 5 min of stable sweating at low‐ and moderate‐heat strain, were compared between groups (young, older) and regions (trunk, limbs) within each level of heat strain using a linear mixed‐effects model with nested intercepts (sites nested within region nested within participant). At low‐heat strain, the age‐related reduction in sweat rate (older‐young values) was greater at the trunk (0.65 mg/cm^2^/min [95% CI 0.44, 0.86]) compared to the limbs (0.42 mg/cm^2^/min [0.22, 0.62]; interaction: *p *= 0.010). At moderate‐heat strain, sweat rate was lower in older compared to young (main effect: *p *= 0.025), albeit that reduction did not differ between regions (interaction: *p *= 0.888). We conclude that, contrary to previous suggestions, the age‐related decline in sweat rate was greater at the trunk compared to the limbs at low‐heat strain, with no evidence of regional variation in that age‐related decline at moderate‐heat strain.

## Graphical abstract

Sweat rate was measured at six sites across the limbs (*n* = 4) and trunk (*n* = 2) in young and older adults at controlled elevations in esophageal temperature of 0.6°C (low‐heat strain) and 1.2°C (moderate‐heat strain) above baseline resting to evaluate the regional age‐related decline in sweating. At low‐heat strain, sweat rate was attenuated in older adults to a greater extent at the trunk than the limbs, with no evidence of that regional pattern in the age‐related reduction observed at moderate‐heat strain.

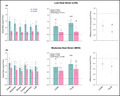

## 1 INTRODUCTION

Older adults (aged ≥65 years) are among the most vulnerable to the adverse health effects of heat exposure (Meade et al., 2020), due at least in part to age‐related decrements in sudomotor function (Inoue & Shibasaki, 1996; Shibasaki et al., 2013). These include a delay in the onset of sweating and a delay in sweat gland output, which reduces sweat rate relative to young adults (aged 18–30 years) at a given increase in mean body temperature (Inoue & Shibasaki, 1996; Sato & Sato, 1983). Interestingly, some investigations have reported that the magnitude of this reduction is greater at the limbs compared to the trunk (Coull et al., 2021; Inoue et al., 1991; Inoue & Shibasaki, 1996; Kenney & Munce, 2003), leading to the suggestion that age‐related decrements in sweat rate may develop in a peripheral‐to‐central manner (Inoue et al., 1991; Kenney & Munce, 2003). However, subsequent investigations of this hypothesis are equivocal, with some providing supporting evidence, whereas others have observed a non‐region‐specific pattern of reduction (Gerrett et al., 2021; Smith et al., 2013).

Inconsistencies with previous observations may relate to differences in the experimental protocols employed, which have included walking on a treadmill in the heat (Coull et al., 2021) or lower‐limb water immersion (Gerrett et al., 2021; Inoue et al., 1991; Inoue & Shibasaki, 1996). The former resulted in higher skin temperatures at the trunk and lower limbs compared to the upper limbs in older adults (Coull et al., 2021), an observation likely due to the increased heat production from the active musculature in the lower limbs (Kenny & Flouris, 2014; Todd et al., 2014). This makes it difficult to discern the true magnitude of any regional variation in the age‐related decline in sweating, and whether regional variations in that decline reflect a peripheral‐to‐central pattern or simply reflect segmental differences in skin temperature, which can independently modulate sweat rate (Nadel et al., 1971). While those investigators employing lower‐limb hot‐water immersion did not report regional skin temperature (Gerrett et al., 2021; Inoue et al., 1991; Inoue & Shibasaki, 1996), it is likely that a similar regional difference occurred given the upper body was exposed to warm air, which has markedly lower thermal conductivity. Further, since both models elicited a single step change in body core temperature of ≤0.8°C, it remains uncertain as to whether regional variations in the age‐related reduction in sweating (if any) occur at higher levels of heat strain.

To our knowledge, the only study employing a different approach was Smith et al. (2013), who utilized a water‐perfused suit covering most of the body surface to raise body core temperature 0.5 and 1.0°C above resting levels. This model ensures a more homogenous skin temperature response across measurement regions, while also allowing sweat rate to be assessed at two body core temperatures that were matched between young and older adults. However, this report, along with others (Gerrett et al., 2021; Inoue, 1996; Inoue et al., 1991), included measures of sweat rate from a single region on the upper (forearm) and lower (quadriceps) limbs and two sites on the trunk (abdomen and lower back), which may not accurately reflect sweat rate at the periphery due to the extensive inter‐ and intra‐regional variation in sweat rate (Taylor, & Machado‐Moreira., 2013).

The purpose of the present study was therefore to evaluate sweat rate during passive heat stress eliciting controlled elevations in body core and skin temperatures between young and older adults, as an exploratory extension of our recent work on regional variation in the reliability of sweat rate measured using the ventilated capsule technique (Rutherford et al., 2021). To ensure regional sweat rates were not overly influenced by one site, sweat rate was measured at four sites on the limbs and two sites on the trunk in young and older adults at two levels of heat strain (0.6 and 1.2°C above baseline). With this unique approach, we were able to provide a more robust test of a peripheral‐to‐central decline in the age‐related reduction in sweat rate, while also determining whether these effects are consistent across two separate levels of heat strain.

## 2 METHODS

### 2.1 Ethical approval

The experiment was approved by the University of Ottawa Health Sciences and Science Research Ethics Board (H‐05‐16‐17) and agrees with the latest revision of the *Declaration of Helsinki*, except for registration in a data base. All participants provided written informed consent prior to participating. All experiments took place at the Human and Environmental Physiology Research Unit located at the University of Ottawa.

### 2.2 Participants

Fifteen older (mean (SD); 69 (4) years) and 14 young men (24 (5) years) participated (Table 1). Only participants who were healthy (free of any cardiovascular, respiratory, autonomic or metabolic conditions), non‐smokers, and not taking prescription medication were eligible to participate in the current study. Further, all participants were habitually active (i.e., performed ≥30 min of structured physical activity at least twice per week) as determined by a standardized questionnaire (Baecke et al., 1982). Data from the young men have been reported as part of a larger project assessing the reliability of microvascular, sudomotor, and cardiovascular autonomic function (Akerman et al., 2021; Gemae et al., 2021; Rutherford et al., 2021). Given the exploratory nature of the study, no a priori power analysis was performed.

**TABLE 1:** Physical characteristics of the young (*n* = 14) and older (*n* = 15) men.

	Age (years)	Height (cm)	Mass (kg)	*A_D_* (m^2^)	Body fat (%)	V̇O_2_peak (ml/min/kg)
Young						
Mean (SD)	24 (5)	176 (5)	79.9 (12.3)	2.0 (0.2)	15.8 (5.7)	43.4 (8.3)
Min–max	18–32	168–184	65.1–102.3	1.7–2.3	8.0–28.0	33.0–61.4
Older						
Mean (SD)	69 (4)	171 (6)	74.9 (9.7)	1.9 (0.1)	21.1 (4.8)	33.0 (7.5)
Min–max	65–77	160–182	58.7–94.1	1.6–2.1	14.6–24.7	19.5–51.4
*p* value	<0.001*	0.029*	0.235	0.083	0.001*	0.002*

Note

*p* values denote results from an unpaired, two‐tailed *t*‐test.

Abbreviations: *A*_D_, body surface area; V̇O_2peak_, peak oxygen consumption.

* Significantly different from the young; *p *< 0.05.

### 2.3 Experimental design

Participants completed one screening session and one experimental session in a temperature‐controlled laboratory (~25°C). The experimental sesssion for the young group was the first of three sessions reported in our previous work (Akerman et al., 2021; Gemae et al., 2021; Rutherford et al., 2021). The firstexperimental session for the young group was chosen as it provided the most naïve assessment between both age groups. The experimental session for the older adults were performed during the northern hemisphere fall and winter months (September–February) whereas the young group completed the experimental session during the southern hemisphere summer and fall months (June–November). Prior to each session, participants were instructed to abstain from heavy exercise and alcohol for ≥24 h, caffeine for at least 4 h, and food consumption for ≥2 h.

### 2.4 Screening session

Standing height, body mass, body surface area, and body fat percentage, as well as peak oxygen consumption were determined during the preliminary visit. Body surface area was derived from measures of standing height (model 2391; Detecto) and body mass (IND560; Mettler Toledo Inc.; DuBois & DuBois, 1915). Hydrostatic weighing was used to estimate body fat percentage (Siri, 1956). The only exceptions were three older adults who had their body fat percentage estimated with bioelectrical impedance (BF‐679W, TANITA Corporation). Peak oxygen consumption was measured (MCD Medgraphics Ultima Series; MGC Diagnostics) during incremental cycle exercise to volitional exhaustion (CSEP, 1986).

### 2.5 Experimental trial

After confirming euhydration (urine specific gravity of ≤1.025; Kenefick & Cheuvront, 2012), participants were instrumented in the supine position before donning a high‐density water perfusion suit (Med‐Eng, Ottawa, Canada) covering the entire body except for the feet, head, and hands, which was perfused with water regulated to 34°C (DC30‐K20 Digitcal Control Bath; Thermo Scientific Haake). Participants then reassumed a supine position for ~30 min before baseline data were collected for 10 min (no‐heat strain [NHS]). Water bath temperature was then increased to 49.5°C and participants were covered up to the neck with a plastic sheet, fleece blanket, and two natural runner mats to increase esophageal temperature by 0.6°C (low‐heat strain [LHS]) and 1.2°C (moderate‐heat strain [MHS]) above baseline. At each level of heat strain, water bath temperature was reduced to 43°C when esophageal temperature was 0.1°C below the target temperature to elicit a plateau. Once esophageal temperature and sweat rate had stabilized, data were collected for 10 min before increasing water temperature to 49.5°C to the next level of heat strain.

### 2.6 Measurements

Esophageal temperature was measured using a thermocouple probe inserted ~40 cm past the nares (Mon‐a‐therm general purpose temperature probe; Mallinckrodt Medical) and recorded at 15‐s intervals (HP Agilent data‐acquisition module, model 2497A or Powerlab; ADInstruments). Skin temperature was measured at 1‐min intervals using digital thermometers (iButton DS1921H‐F5#; Maxim Integrated Products) affixed to the chest, abdomen, biceps, forearm, quadriceps, and calf (~1 cm adjacent to each ventilated capsule) using tape (Transpore; 3 M). Mean skin temperature was approximated from a weighted average of four sites (biceps: 30%, chest: 30%, quadriceps: 20%, calf: 20%; Ramanathan, 1964). Mean body temperature was estimated as the weighted average of esophageal (80%) and mean skin temperature (20%) (Hardy et al., 1938).

**FIGURE 1:**
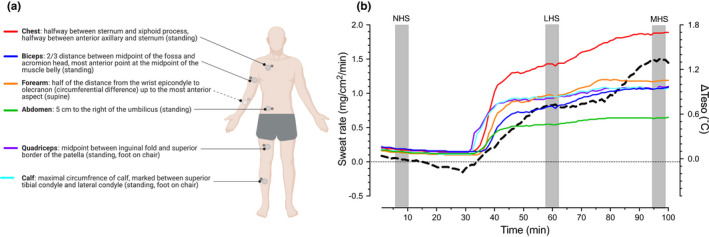
(a) Visual representation of measurement locations for local sweat rates (large light grey circles) measured via ventilated capsules and skin temperature (small dark grey circles) measured via iButtons with the corresponding landmarking instructions used for the placement of the capsules and colored lines matching the line colors in panel b (created using BioRender.com). (b) Representative profile of sweating demonstrating the periods used in data analysis. LSR, local sweat rate; Δ*Teso*, change in esophageal temperature (black‐dashed line); NHS, no‐heat strain; LHS, low‐heat strain; MHS, moderate‐heat strain. Adapted with permission from Rutherford et al. (2021).

Local sweat rate was measured at six sites (chest, abdomen, biceps, forearm, quadriceps, calf) using 3.8 cm^2^ plastic ventilated capsules affixed to the skin using adhesive rings and topical skin glue (Collodion HV; Mavidon Medical Products), and covered by custom‐made perforated plastic dome‐shaped shields (~37 cm^2^) to minimize temperature and pressure artefacts (Frei et al., 2019). A landmarking guide was used to standardize capsule placement (Figure 1a). Dry, compressed air was passed through each capsule at a rate of 0.75 L/min (Omega FMA‐A2307; Omega Engineering). The absolute humidity of effluent air from each sweat capsule was measured with capacitance hygrometers (model HMT333; Vaisala), which were calibrated according per manufacturer's specifications with standard salt solutions (LiCl, 11% relative humidity; NaCl, 75% relative humidity) prior to experimentation (HMK15 Humidity Calibrator; Vaisala). Local sweat rate was calculated every 5 s using the differences in the absolute humidity of effluent and influent air multiplied by the flow rate and normalized to the encapsulated skin surface area (mg/cm^2^/min).

The following equation was used to calculate local sweat rate (in mg/cm^2^/min):

$$\text{Sweat rate}\;=\frac{\text{Absolute humidity}\times \text{Flow rate}}{\text{Surface area}},$$

where flow rate is the rate of air flow through the capsules in L/min, surface area was the area of skin covered by the sweat capsule in cm^2^ and the absolute humidity of effluent air, in g/m^3^ was determined by:

$$\begin{array}{c} \text{Absolute humidity}=\\ \;2.17\times \frac{\text{Saturated water vapor pressure}\times \text{Relative humidity}}{\text{Air temperature}+273.15}, \end{array}$$

where the saturated water vapor pressure is the partial pressure of water vapor in fully saturated air in kPa, relative humidity is the % saturation of effluent air leaving the capsules with water, air temperature is the temperature of effluent air in °C, and 273.15 is the conversion from °C to °K.

Data were recorded with LabVIEW software (version 7.0; National Instruments). Sweat rates at each site were normalized to the individual NHS values to account for baseline variation.

### 2.7 Data analysis

Local sweat rates, esophageal, mean skin, and mean body temperatures were expressed as minute averages, with an average of the final 5 min of each 10 min data collection period being used for the statistical analysis (Figure 1b). The onset threshold for the activation of sweating was determined as the mean body temperature (derived at 1 min intervals) at which a sustained increase in sweat rate of >0.05 mg/cm^2^/min occurred (Patterson et al., 2004). The thermosensitivity (slope) of the relationship between sweat rate and mean body temperature after the onset was determined using linear regression.

### 2.8 Statistical analysis

Physical characteristics and heating time within each stage were compared between‐groups using unpaired, two‐tailed *t*‐tests. Body temperature data were compared using a linear mixed‐effects model with the fixed factors of group (young, older) and heating stage (NHS, LHS, MHS). Random effects (random intercept or intercept and slope) and variance/covariance structures were determined using Akaike's information criterion. When a significant interaction was detected, *post*‐*hoc* comparisons were carried out using Bonferroni‐adjusted unpaired (group) and paired (stage), two‐tailed *t*‐tests. Sweating onset, thermosensitivity, and local sweat rate within LHS and MHS were compared using linear mixed effects models with the fixed factors of group (young, older) and region (trunk, limbs) and a nested random intercept (site within region within participant), with a significant interaction indicating a difference in the magnitude of the age‐related decrement between the trunk and limbs. Local skin temperature at each site was included as a covariate to account for any between‐group and between‐region variations in skin temperature. As a secondary component, we explored whether differences in the age‐related change in sweating exist between‐ and within‐body segments at these six sites under two levels of heat strain (LHS, MHS). To achieve this, a mixed‐effects model with the fixed effects of group and site (six levels: chest, abdomen, biceps, forearm, quadriceps, calf) and the same nested intercept as mentioned above was used.

Test assumptions of normality, linearity and homoscedasticity were confirmed by inspecting quantile‐comparison, scatter and residual plots. *Alpha* was set at 0.050, with data being reported as mean (SD) unless stated otherwise as mean [95% CI]. *p*‐values for the exploratory comparisons of sweat rate between sites were corrected using the Bonferroni procedure. All analyses were performed using R (Version 3.6.1; Lenth et al., 2018; Pinheiro et al., 2017; R Core Team, 2018).

## 3 RESULTS

Compared to the young, the older group were shorter, possessed a higher body fat percentage, and had a lower peak oxygen consumption (all *p *≤ 0.029; Table 1). Body mass and surface area did not differ between groups (both *p *≥ 0.083; Table 1). Heating time was not different between the young and older adults at LHS (50 (5) and 52 (6) min, respectively; *p* = 0.287) and at MHS (84 (11) and 85 (9) min, respectively; *p* = 0.657).

Body temperature data are presented in Table 2. Esophageal temperature increased with heating stage across groups (main effect: *p *< 0.001) and was higher in young compared to older adults across stages due to a higher resting body core temperature (main effect: *p* = 0.001). The change in esophageal temperature increased with heating stage (main effect: *p *< 0.001), but it did not differ between groups (main effect: *p* = 0.142). Mean skin temperature and mean body temperature differed as a function of group and heating stage (interaction: all *p *< 0.001), such that they were higher in young compared to older adults at all stages (both *p* ≤ 0.001). Trunk skin temperature (interaction: *p* = 0.001) was lower in the older adults at NHS and MHS (both *p *≤ 0.023) but was not different between groups at LHS (*p* = 0.312). Limb skin temperature increased with heating stage (main effect: both *p *< 0.001) and was higher in the young compared to older adults (main effect: both *p *< 0.001).

**TABLE 2:** Duration of heating, esophageal temperature, the change in esophageal temperature, mean skin temperature, and mean body temperature at all three heating stages in young (*n* = 14) and older (*n* = 15) men

	Young, mean (SD)	Older, mean (SD)	Diff. from young, mean [95% CI]	*p‐*values		
				Condition	Group	Interaction
Esophageal temperature (°C)						
No‐heat strain	36.9 (0.2)	36.5 (0.4)	−0.4 [−0.7, −0.2]*	<0.001	0.001	0.317
Low‐heat strain	37.5 (0.2)	37.1 (0.3)	−0.4 [−0.6, −0.1]*			
Moderate‐heat strain	38.1 (0.2)	37.7 (0.4)	−0.3 [−0.6, −0.1]*			
∆ Esophageal temperature (°C)						
No‐heat strain				<0.001	0.142	0.487
Low‐heat strain	0.6 (0.1)	0.7 (0.1)	0.1 [−0.1, 0.2]			
Moderate‐heat strain	1.2 (0.1)	1.3 (0.2)	0.1 [0.0, 0.2]			
Mean skin temperature (°C)						
No‐heat strain	34.9 (0.5)	34.0 (0.6)	−1.0 [−1.4, −0.5]*	<0.001	<0.001	0.008
Low‐heat strain	37.6 (0.3)	37.3 (0.4)	−0.3 [−0.6, 0.0]*			
Moderate‐heat strain	38.1 (0.4)	37.7 (0.3)	−0.4 [−0.7, −0.1]*			
Trunk skin temperature (°C)						
No‐heat strain	35.2 (0.7)	34.1 (1.0)	−1.1 [−1.7, −0.4]	<0.001	0.243	0.001
Low‐heat strain	37.6 (0.3)	37.5 (0.2)	−0.1 [−0.3,0.1]			
Moderate‐heat strain	38.2 (0.4)	37.7 (0.6)	−0.5 [−0.9, −0.1]			
Limbs skin temperature (°C)						
No‐heat strain	34.4 (0.6)	33.6 (0.7)	−0.8 [−1.3, −0.3]*	<0.001	<0.001	0.254
Low‐heat strain	37.5 (0.3)	37.1 (0.5)	−0.4 [−0.7, −0.1]*			
Moderate‐heat strain	38.1 (0.3)	37.7 (0.4)	−0.4 [−0.7, −0.1]*			
Mean body temperature (°C)						
No‐heat strain	36.5 (0.3)	36.0 (0.4)	−0.5 [−0.8, −0.3]*	<0.001	<0.001	0.006
Low‐heat strain	37.5 (0.2)	37.2 (0.3)	−0.4 [−0.6, −0.1]*			
Moderate‐heat strain	38.1 (0.3)	37.7 (0.4)	−0.4 [−0.6, −0.1]*			

Notes

Mean skin temperature was calculated as a weighted average (arm: 30%, chest: 30%, biceps: 20%, quadriceps: 20%; Ramanathan, 1964). Mean body temperature was estimated from the weighted sum of esophageal (0.8) and mean skin temperature (0.2) (Hardy et al., 1938). Trunk skin temperature represents the unweighted average of skin temperature on the chest and abdomen. Limbs skin temperature represents the unweighted average of skin temperature on the biceps, forearm, quadriceps, and calf. *p‐*values are provided for a linear mixed‐effects model with the non‐repeated factor of group (young, older) and repeated factor of condition (no‐heat strain, low‐heat strain, moderate‐heat strain).

* Significantly different from the young; *p *< 0.050.

**FIGURE 2:**
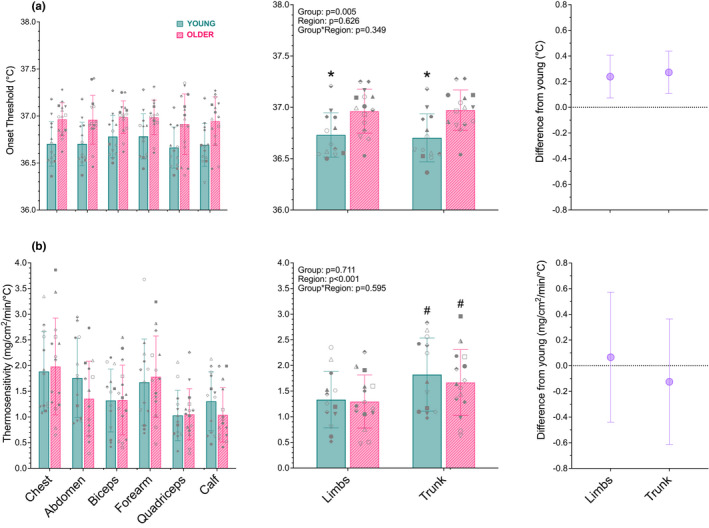
The onset threshold (panel a) and thermosensitivity (panel b) of sweating during whole‐body passive heating in young (*n* = 14; blue, solid) and older (*n* = 15; pink, dashed) men at the individual sites (left panel) and by region (middle panel; trunk: chest, abdomen; limbs: biceps, forearm, quadriceps, calf). All data are presented as mean (SD) with individual values overlayed in the panels on the left and middle with the associated mean difference [95% CI] in regional onset threshold and thermosensitivity between the older and young group on the right panels. *p*‐values are provided for a linear mixed‐effects model with nested intercepts (sites nested within region nested within participant), and with skin temperature at each site included as a covariate. *, denotes a significantly earlier onset compared to the older adults (*p* < 0.050); #, denotes significantly greater than the limbs (*p* < 0.050)

The onset and thermosensitivity of the sweating response are presented in Figure 2. The onset of sweating was 0.3°C [0.1, 0.4] higher in the older compared to young (main effect: *p* = 0.004), but it did not differ between regions (main effect: *p* = 0.626). Further, the age‐related delay in the onset threshold for sweating was not region‐dependent (interaction: *p* = 0.349). The thermosensitivity of the sweating response was 0.44 mg/cm^2^/min/°C [0.25, 0.63] higher at the trunk compared to the limbs (main effect: *p* = 0.001) but did not differ between groups (main effect: *p* = 0.711). The region‐related difference in thermosensitivity was not influenced by age (interaction: *p* = 0.595).

**FIGURE 3:**
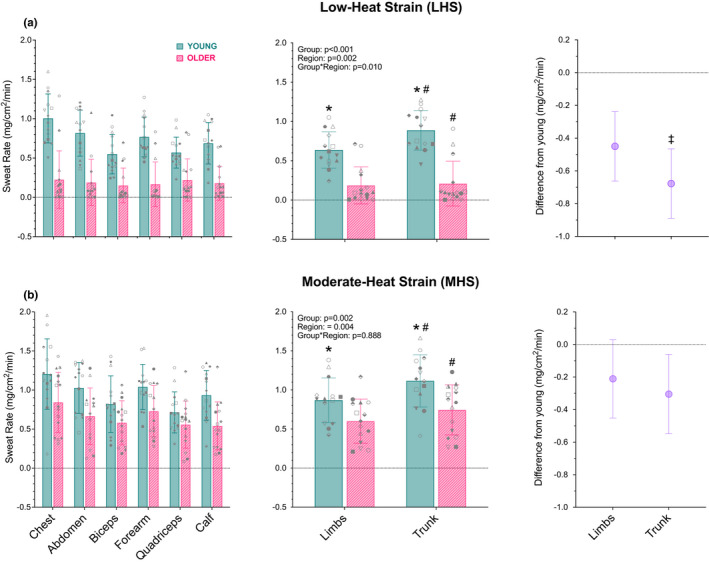
Local sweat rate at low‐heat strain (LHS; panel a) and moderate‐heat strain (MHS; panel b) in young (*n* = 14; blue, solid) and older (*n* = 15; pink, dashed) men at the individual sites (far left panels) and the trunk and limb regions (middle panels; trunk: chest, abdomen; limbs: biceps, forearm, quadriceps, calf) during whole‐body passive (resting) heating to raise and clamp esophageal temperature 0.6°C (low heat strain) and 1.2°C (moderate heat strain) above baseline. Local sweat rate is presented as a mean (SD) with individual values overlayed in the panels on the left and middle with the associated mean difference [95% CI] in regional sweating between the older and young group on the right panels. *p* values are provided for a linear mixed‐effects model with nested intercepts (sites nested within region nested within participant), and skin temperature at each site included as a covariate. *, denotes significantly greater than older adults; #, denotes significantly greater than the limbs (*p* < 0.050); ‡, denotes significantly greater age‐related difference versus the limbs (*p* < 0.050)

The individual and the averaged sweat rates at LHS and MHS are presented in Figure 3. A group‐by‐region interaction at LHS was observed (*p *< 0.010), indicating that sweat rate was reduced in older compared to young adults to a greater extent at the trunk by 0.65 mg/cm^2^/min [0.44, 0.86] compared to the limbs (0.42 mg/cm^2^/min [0.22, 0.62]). At MHS, sweat rate did not differ as a function of group and region (interaction: *p* = 0.888) but it was reduced by 0.34 mg/cm^2^/min [0.13, 0.54] in older compared to young adults across regions (main effect: *p* = 0.025), and lower by 0.17 mg/cm^2^/min [0.06, 0.27] at the limbs relative to the trunk across groups (main effect: *p* = 0.001).

**FIGURE 4:**
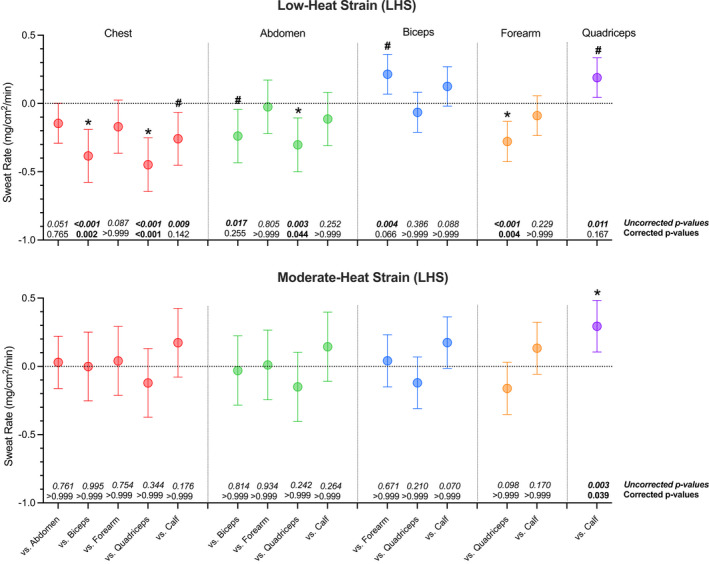
Between site difference in the age‐related decline in sweat rate between individual sites at the chest (red), abdomen (green), biceps (blue), forearm (orange), and quadriceps (purple) during whole‐body passive (resting) heating to raise and clamp esophageal temperature 0.6°C (low heat strain, LHS, panel a) and 1.2°C (moderate heat strain, MHS; panel b) above baseline in young (*n* = 14) and older (*n* = 15) men are presented on the panels on the left. For example, the difference in the age‐related change at the chest vs. the abdomen (left most comparison) was calculated as (abdomen older−abdomen young)−(chest older−chest young). On the bottom of panel a and b, the corresponding uncorrected *p*‐values and corrected *p*‐values using Bonferroni‐adjustments are provided. All data are reported from a linear mixed‐effects model with the fixed effects of group and site (six levels: chest, abdomen, biceps, forearm, quadriceps, calf) with nested random intercepts (sites nested within region nested within participant), and skin temperature at each site included as a covariate. *, denotes a significant difference (*p* < 0.050) when *p*‐values are uncorrected; #, denotes a significant difference (*p* < 0.050) when p‐values are corrected using Bonferroni‐adjustments.

The individual site analysis at LHS and MHS are presented in Figure 4. At LHS, a group‐by‐site interaction for sweat rate was observed (*p *< 0.001). When comparing between body segments, a greater age‐related reduction in sweat rate at the chest and abdomen compared to the biceps and quadriceps (all *p *≤ 0.017) was observed. When comparing within body segments, the age‐related change in sweat rate was lower at the biceps (0.21 mg/cm^2^/min [0.06, 0.36], *p* = 0.004) and quadriceps (0.19 mg/cm^2^/min [0.04, 0.33], *p* = 0.011) compared to the forearm and calf, respectively. At MHS, sweat rate did not differ as a function of group and site (group‐by‐site interaction: *p* = 0.089), however, the age‐related change in sweat rate was still lower at the quadriceps (0.29 mg/cm^2^/min [0.011, 0.48], *p* = 0.003) compared to the calf.

## 4 DISCUSSION

In this report, we revaluated the hypothesis that age‐related decrements in sweat rate may be more pronounced at the limbs compared to the trunk. This was achieved by measuring sweat rate at six sites across the trunk and limbs in young and older adults during whole‐body passive heat stress to increase and clamp esophageal temperature by 0.6°C (low‐heat strain) and 1.2°C (moderate‐heat strain) above baseline. Contrary to previous suggestions, sweat rate was attenuated in older relative to young men to a greater extent at the trunk compared to the limbs at low‐heat strain, with no evidence of regional variation in that age‐related reduction during moderate‐heat strain. These outcomes therefore advance our knowledge by indicating that aging may compromise sweat rate to a greater extent at central compared to peripheral regions during low‐, but not moderate‐heat strain, perhaps due to regional differences in sweat rate and/or the need to preserve sweating at areas with the greatest capacity for heat loss.

Age‐related decrements in sweat rate have previously been suggested to develop in a peripheral‐to‐central manner (Inoue et al., 1991; Kenney & Munce, 2003). However, previous investigations of this hypothesis employed experimental models eliciting regional variations in skin temperature (Coull et al., 2021; Gerrett et al., 2021; Inoue et al., 1991; Inoue & Shibasaki, 1996) or measured sweat rate at a single region on the upper and lower limbs (Inoue, 1996). We therefore revisited this hypothesis by assessing sweat rate in young and older adults at four sites on the limbs to reflect the periphery and two sites on the torso to reflect the trunk (central). Contrary to the peripheral‐to‐central age‐related decline documented previously (Inoue et al., 1991), we observed a greater reduction in sweat rate at the trunk compared to the limbs in the older relative to young adults at low‐heat strain (Figure 3a). While there is a need for larger and more detailed studies to identify the mechanism(s) explaining this observation and the discrepancy between our findings and those of previous reports, this outcome advances understanding by providing evidence for a central‐to‐peripheral age‐related reduction in sweating and at a minimum, indicates that previously held beliefs should be re‐examined with more rigorous control of regional variations in skin temperature.

Our study was not designed to identify the physiological significance of the observed regional difference at low‐heat strain (Figure 3a); however, this may partly reflect regional differences in sweat rate and/or the need to preserve sweating at areas with the greatest capacity for heat loss. The trunk is typically associated with higher sweat rates than the limbs (Taylor, & Machado‐Moreira, 2013) and this was confirmed in the present study (Figure 3). It is possible, therefore, that aging impacts sweat rate to a greater extent at the trunk compared to the limbs, since this region has a greater capacity to be degraded; a mechanism akin to that associated with sarcopenia (Ata et al., 2019). Further, due to their high surface‐area‐to‐mass ratio, the limbs have a greater relative capacity for evaporative heat loss compared to the trunk (Taylor, & Machado‐Moreira, 2013). Thus, it is plausible that sweating is preserved with aging at the areas with the greatest capacity for heat loss, albeit it remains uncertain as to whether such regional differences can be attributed to functional or structural changes. Nonetheless, these hypotheses remain speculative and represent an important area of future research for improving our understanding of age‐associated changes in thermoregulatory function.

In contrast to the central‐to‐peripheral age‐related decline in sweat rate observed at low‐heat strain, a non‐region‐specific pattern of reduction was observed at moderate‐heat strain (Figure 3b). This may be because sweat rate becomes more homogenous across the body surface with increasing heat strain (Kondo et al., 2001; Taylor, & Machado‐Moreira., 2013), reducing the magnitude of any regional differences in the age‐related decline in sweating. We were unable to confirm this statistically, as indicated by a significant group‐by‐region‐by‐heating stage interaction, due to the large sample size required to perform a meaningful evaluation of such an effect (Heo & Leon, 2010). However, our findings are consistent with previous investigations employing models eliciting higher levels of heat strain (~0.8–1.0°C; Gerrett et al., 2021; Smith et al., 2013), and indicate that any regional variation in the age‐related decrement in sweat rate may be less apparent at higher levels of heat strain.

A secondary interest for this study was to compare the magnitude of any age‐related decline in sweat rate between the individual sites comprising each region (trunk and limbs). Consistent with when grouped into trunk and limb regions, the chest had the greatest age‐related reduction in sweat rate followed by the abdomen (Figure 4). Since both sites on the trunk (abdomen and chest) displayed a greater age‐related reduction than the biceps, quadriceps, and calf, we can be reasonably certain that the central‐to‐peripheral pattern observed in our primary analysis (Figure 3) was not heavily influenced by one specific site and was region based. Additionally, at moderate‐heat strain, the age‐related reduction in sweat rate was similar between all six sites. This observation further supports the notion that sweat rate becomes more homogenous across the body surface with increasing levels of heat strain.

Our primary interest for this experiment was age and regional differences in sweat rate when body core temperature was raised and clamped (i.e., low‐ and moderate‐heat strain). However, it is important to note that the regional difference in the age‐related decline in sweat rate occurring during low‐heat strain (Figure 3a) was not observed for the thermosensitivity of the sweating response, which did not differ between young and older men (Figure 2b). Further, while the onset of sweating was delayed in older compared to young men, this delay did not differ between the trunk and limbs (Figure 2a). This outcome is in line with previous work where a non‐region‐specific delay in onset threshold was observed in older compared to young adults (Inoue et al., 1991; Inoue & Shibasaki, 1996; Smith et al., 2013). Thus, although aging delays the onset of sweating, our findings support previous suggestions that sweat gland recruitment occurs in a generalized pattern across the body surface during thermal loading, irrespective of age (Frei et al., 2019).

### 4.1 Perspectives

Although our study was directed at advancing our understanding of age‐related changes in thermoregulatory function, our findings may also aid in informing heat‐mitigation strategies to protect older adults, who are particularly vulnerable to the adverse effects of extreme heat (Meade et al., 2020). To reduce the thermal and cardiovascular burden of heat stress, various low‐cost heat‐mitigation strategies have been proposed including misting and water dousing (Morris et al., 2019), which serve to enhance evaporative cooling by wetting the skin. Such strategies may be particularly beneficial in older adults during hot‐dry environments, due to their lower capacity for evaporative heat loss relative to young adults (Kenny et al., 2017). Given we observed a greater age‐related decrement in sweat rate at the trunk compared to the limbs during low‐heat strain (Figure 3), it is possible that older adults could maximize any benefit of these strategies by placing greater emphasis on wetting the trunk, as opposed to the limbs. Further, during moderate‐heat strain, no evidence of regional variation in that age‐related reduction occurred. Therefore, heat‐mitigation strategies that aim to cool the whole‐body, such as ensuring access to central air conditioning and urban redesign (e.g., cooling roofs) can improve thermal comfort in individuals when exposed to extreme heat (Broadbent et al., 2021; Quinn et al., 2017).

### 4.2 Considerations

There are several noteworthy considerations associated with the current study. First, activated sweat gland density was not measured in the current study. We were therefore unable to calculate sweat gland output and determine if age‐related declines in sweat rate were due to reduced sweat gland output and/or fewer active glands. Second, we did not assess potential modulators for the full expression of sweating such as nitric oxide, which are known to be diminished in older adults (Amano et al., 2017; Stapleton et al., 2014), which may aid in explaining regional variations in the age‐related decline in sweat rate. Third, despite possessing a lower peak oxygen consumption (V̇O_2peak_) than their younger counterparts (Table 1), the older men in the current study would still be considered as relatively well‐trained, with a V̇O_2peak_ in the 95th percentile of age‐ and sex‐specific normative values (Hoffmann et al., 2019). Increased aerobic fitness, as indexed by V̇O_2peak_, can attenuate age‐related reductions in evaporative heat loss during exercise‐heat stress (Notley et al., 2020a), which in turn may have reduced age‐related reductions in sweat rate and the magnitude of regional variation in that decrement compared to less aerobically fit older adults. Fourth, we assessed the older and young adults in different seasons. It is possible that the age‐related differences observed may have been slightly inflated due to seasonal acclimatization in the young adults. However, we anticipate this effect to be relatively small based on our previous work demonstrating comparable thermoregulatory function in young adults prior to and following summer (Notley, Meade, Akerman, et al., 2020). Fifth, we did not assess the effect of posture on the regional age‐related decline in sweat rate. In young adults, regional variations in sweating are modified by posture (seated and supine; Inukai et al., 2005; Shim & Choi, 1993), which is likely due to variations in skin pressure (i.e., when seated the thigh sweat rate is reduced relative to the forearm due to greater skin pressure on the lower limbs). Therefore, postural differences between studies may partly explain the inconsistencies in results within the existing literature assessing the regional age‐related decline in sweat rate (Coull et al., 2021; Gerrett et al., 2021; Inoue et al., 1991; Inoue & Shibasaki, 1996; Kenney & Munce, 2003), and this represents an important area of future research. Finally, given our data are restricted to males, there is a need for further studies to determine whether sex modulates regional differences in the age‐related decline in sweating. However, since evaporative heat loss during exercise‐heat stress may decline at a similar rate with increasing age in males and females (D'Souza et al., 2020), we would expect similar observations between sexes.

## 5 CONCLUSION

It has been suggested that age‐related decrements in sweat rate may be more pronounced at the limbs compared to the trunk. However, we demonstrate that, when assessed at multiple regions on the skin surface of these areas during passive heating eliciting 0.6°C (low‐heat strain) and 1.2°C (moderate‐heat strain) elevations in body core temperature, sweat rate was attenuated in older compared to young adults to a greater extent at the trunk relative to the limbs at low‐heat strain, but not moderate‐heat strain. While larger, confirmatory studies are needed, these outcomes indicate that age‐related reductions in sweat rate may develop in a central‐to‐peripheral direction, albeit with an important role for the level of heat strain at which sweat rate is assessed.

## CONFLICT OF INTEREST

No conflicts of interest, financial or otherwise, are declared by the author(s).

## AUTHOR CONTRIBUTIONS

All authors conceived and designed of the work, revised the manuscript, approved the final version of the manuscript, and agree to be accountable for all aspects of the work. M.D.S., S.R.N., A.P.A., R.D.M., and M.M.R. performed data collection and analysis. M.D.S., S.R.N., and R.D.M., performed statistical analysis. M.D.S and S.R.N drafted the manuscript. M.D.S and S.R.N prepared figures. All persons designated as authors qualify for authorship, and all those who qualify for authorship are listed.
